# The burden of chronic pain for patients with osteoarthritis in Germany: a retrospective cohort study of claims data

**DOI:** 10.1186/s12891-021-04180-1

**Published:** 2021-03-31

**Authors:** Marie Schild, Ulrike Müller, Ursula von Schenck, Sigurd Prieur, Robert Miller

**Affiliations:** 1 Pfizer Germany GmbH, Berlin, Germany; 2 Pfizer Pharma GmbH, Linkstr. 10, 10785 Berlin, Germany; 3 Elsevier Health Analytics, Berlin, Germany

**Keywords:** Health insurance, Health care costs, Cost of illness, Direct service costs, Sick leave

## Abstract

**Background:**

Osteoarthritis (OA) is a common condition that is often associated with chronic pain. Pain often leads patients to seek healthcare advice and treatment. In this retrospective cohort analysis of German longitudinal healthcare claims data, we aimed to explore the healthcare resource utilisation (HRU) and related healthcare costs for patients with OA who develop chronic pain.

**Methods:**

Patient-level data was extracted from the German Institut für Angewandte Gesundheitsforschung (InGef) database. Insured persons (≥18 years) were indexed between January 2015 and December 2017 with a recent (none in the last 2 years) diagnosis of OA. HRU and costs were compared between patients categorised as with (identified via diagnosis or opioid prescription) and without chronic pain. Unweighted HRU (outpatient physician contacts, hospitalisations, prescriptions for physical therapy or psychotherapy, and incapacity to work) and healthcare costs (medication, medical aid/remedy, psychotherapy, inpatient and outpatient and sick pay in Euros [quartile 1, quartile 3]) were calculated per patient for the year following index. Due to potential demographic and comorbidity differences between the groups, inverse probability of treatment weighting (IPTW) was used to estimate weighted costs and rate ratio (RR; 95% confidence interval) of HRU by negative binomial regression modelling.

**Results:**

Of 4,932,543 individuals sampled, 238,306 patients with OA were included in the analysis: 80,055 (34%) categorised as having chronic pain (24,463 via opioid prescription) and 158,251 (66%) categorised as not having chronic pain. The chronic pain cohort was slightly older, more likely to be female, and had more comorbidities. During the year following index, unweighted and IPTW-weighted HRU risk and healthcare costs were higher in patients with chronic pain vs those without for all categories. This led to a substantially higher total annual healthcare cost ─ observed mean; €6801 (1439, 8153) vs €3682 (791, 3787); estimated RR = 1.51 (1.36, 1.66).

**Conclusions:**

German patients with chronic pain and OA have higher healthcare costs and HRU than those with OA alone. Our findings suggest the need for better prevention and treatment of OA in order to reduce the incidence of chronic pain, and the resultant increase in disease burden experienced by patients.

**Supplementary Information:**

The online version contains supplementary material available at 10.1186/s12891-021-04180-1.

## Background

Osteoarthritis (OA) is a common, chronic condition, primarily characterised by joint pain and impaired function that can lead to decreased quality of life for patients. The World Health Organization places OA among the top 10 most disabling diseases in developed countries, with 80% of patients experiencing limitations in movement and 25% left unable to perform their major daily activities [1]. Worldwide, OA is the most prevalent chronic joint disease, estimated to be symptomatic in around 10% of men and 18% of women aged ≥60 years [1, 2]. A 2014 study of German claims data suggested that the prevalence of hip and knee OA was 22% in patients aged ≥60 years (70% of patients were female) [3]. OA is often described as a disease of ‘wear and tear’; as populations around the world grow older and some present with increased obesity, the number of people affected by OA is likely to rise [2, 4, 5]. Despite age being a strong risk factor for OA, many working age patients are also affected ─ more than half of patients with symptomatic knee OA were aged < 65 years in the 2007/8 United States National Health Interview Survey [5]. Early disease is typically associated with comparatively minor functional impairment that may increase over time. A major determinant of functional disability is pain. In late- and end-stage OA, significant functional impairment is often seen in patients with chronic, persistent pain; however, pain is not exclusively attributable to disease progression or structural joint damage, as determined by imaging [6, 7].

Besides non-pharmacological therapies and patient-centred care (considering each patient’s comorbidity profile, goals, expectations, clinical, emotional, and environmental factors, etc.), most patients with OA receive pharmacological therapies. Recommendations for pharmacological therapies in international treatment guidelines for hip and knee OA have subtly evolved over time, generally moving away from paracetamol (acetaminophen) and more clearly defining the situations where opioids might be appropriate [8–12]. This is also the case in German treatment guidelines [13–15]. Joint replacement is generally considered when pain and structural damage becomes intolerably limiting for a patient and cannot be effectively addressed by other therapies [13, 14, 16–18]. In Germany, intolerable pain, loss of function, and structural joint damage evident on radiographs form the indications for joint replacement surgery [13–15, 18]. Yet, invasive joint surgery has inevitable risks and drawbacks, and is not appropriate for all patients, since for some, surgery involves lengthy recovery or can be associated with a risk of persistent post-surgical pain (~ 9% after hip replacement and 20% after knee replacement) [19]. Additionally, although the majority of modern replacement hips and knees have been found to last at least 25 years [20, 21], revision surgery may be required during the lifespan of the patient. Early and effective treatment of OA can contribute to a delay in, or in some cases even prevent, the need for joint replacement.

To date, the incremental socioeconomic burden of chronic pain caused by OA in Germany has not been established. This study used German longitudinal healthcare claims data to quantify the healthcare resource utilisation (HRU) and associated healthcare costs of chronic pain due to OA. The overarching findings from this study are likely generalisable to other countries with similar healthcare systems and demonstrate the substantial costs involved in the attempt to treat patients with OA and chronic pain.

## Methods

This was a retrospective cohort study based on anonymised claims data from the Institut für Angewandte Gesundheitsforschung (InGef) database. This database contains treatment and diagnosis data for more than 4 million insured persons in Germany, stratified by age and gender (as reported by DESTATIS [The Federal Statistics Office]) to ensure adequate representativeness of the German population.

**Fig. 1:**
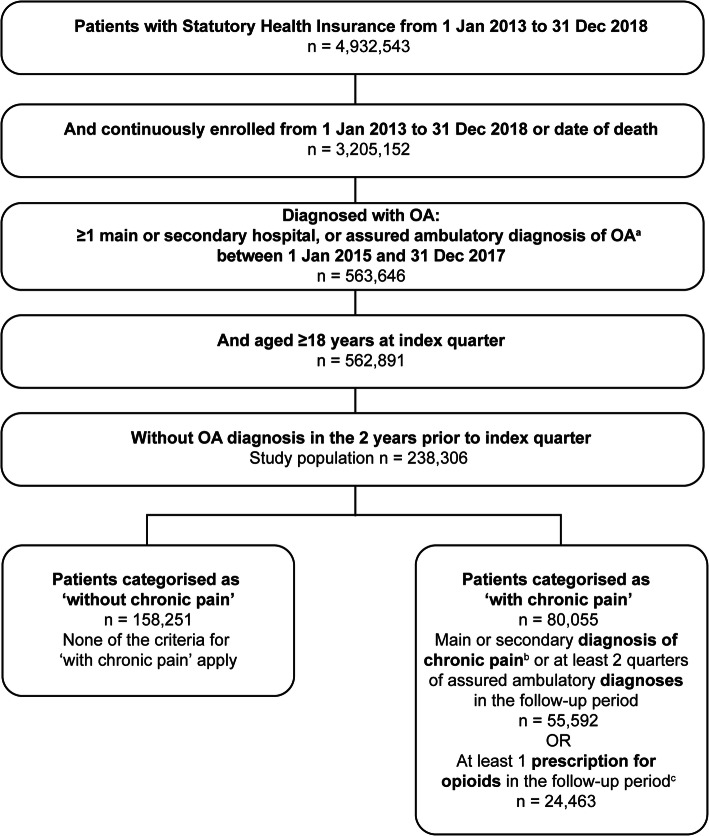
Patient selection. ^a^International Statistical Classification of Diseases and Related Health Problems, 10th edition, German modification (ICD-10-GM) code M16* (hips) or M17* (knee). ^b^ICD-10-GM R52.1, R52.2, F45.41, or F62.80. ^c^Anatomical Therapeutic Chemical Classification System (ATC) code N02AA01, 02, 03, 05, 08, 25, 51, 55, 58, 59, 64, 65, 66, 69, 79, N02AB02, 03, 52, 72, N02AF02, N02AG01, 03, N02AJ01, N02AX01, 02, 05, 51, 52, 62. *OA* Osteoarthritis

Patients ≥18 years of age were indexed on main or secondary hospital or assured ambulatory diagnosis of hip or knee OA (per International Statistical Classification of Diseases and Related Health Problems, 10th edition, German modification [ICD-10-GM] codes M16 [hip] and M17 [knee]) between 01 January 2015 and 31 December 2017 (Fig. 1). Patients must not have had an OA diagnosis in the 2 years prior and must have been continuously enrolled in Statutory Health Insurance from January 2013 to December 2018 (or date of death), allowing for a 2-year baseline period and 1-year follow-up period from the index quarter.

Patients within the study population with chronic pain during the follow-up period were identified by a main or secondary hospital diagnosis of chronic pain (ICD-10-GM codes R52.1, R52.2, F45.41, F62.80), an assured ambulatory diagnoses of chronic pain in at least 2 quarters, or ≥ 1 prescription for a weak or strong opioid (Anatomical Therapeutic Chemical Classification System [ATC] codes N02AA01, 02, 03, 05, 08, 25, 51, 55, 58, 59, 64, 65, 66, 69, 79, N02AB02, 03, 52, 72, N02AF02, N02AG01, 03, N02AJ01, N02AX01, 02, 05, 51, 52, 62; Fig. 1). These patients were categorised as ‘with chronic pain’. All other patients were categorised as being ‘without chronic pain’.

HRU and healthcare costs were compared over the follow-up period for patients categorised as with and without chronic pain. The HRU outcomes were hospitalisations, outpatient physician contacts, incapacity to work, and prescriptions for physical therapy and psychotherapy (Supplementary Table 1, Additional file 1 provides outcome definitions). Model-based estimates of HRU were obtained using negative binomial regression with inverse probability of treatment weighting (IPTW). Healthcare costs were inpatient and outpatient costs, costs for medications, costs for medical aids and remedies, psychotherapy, and sick pay (see Supplementary Table 1, Additional file 1 for definitions). Total costs per patient over the follow-up period were also calculated. All costs were calculated in Euros and modelled on a log scale using linear regression with IPTW to enable relative cost comparisons. Model-implied costs are presented on the original scale.

IPTW was used to adjust for differences in demographics or morbidity profiles in patients categorised as being with and without chronic pain. Weightings were derived from a logistic regression model predicting the probability of being classified as a patient with chronic pain using the following independent variables: type of OA, top 30 most prevalent comorbidities, age, sex, and top 30 most prescribed substances excluding pain medications. The logistic regression coefficients for all variables are shown in Supplementary Table 2, Additional file 2. The distribution of the resulting propensity scores are shown in Supplementary Table 3, Additional file 3; and Supplementary Fig. 1, Additional file 4.

The following formulas were used to calculate the IPTW, where *s* represents the propensity score of the patient, and *p*(*pain*) represents the proportion of patients with chronic pain (i.e. the intercept of a logistic regression model without any covariates).

Patients categorised as with chronic pain:

$$ {w}_{pain}=\frac{p(pain)}{s} $$

Patients categorised as being without chronic pain:

$$ {w}_{no\  pain}=\frac{1-p(pain)}{1-s} $$

The distribution of IPTW was truncated using the 99th percentile as a cut-off point. Based on the accordingly weighted population, logistic regression models were used to estimate the effect of chronic pain on the HRU and cost outcomes, respectively. Rate ratios (RR) with 95% confidence intervals (CIs) were estimated for HRU outcomes.

This study is based on data from the anonymised InGef database and publication was approved by InGef. All analyses were conducted by Elsevier Health Analytics at the Institute for Applied Health Research, Berlin, Germany (InGef). The study was conducted in accordance with all legal and regulatory requirements, as well as with scientific purpose, value, and rigour.

## Results

### Patient characteristics

Of 4,932,543 individuals in the sample, 238,306 patients with OA met the inclusion criteria for the study (Fig. 1). One-third of included patients (34%) were categorised as having chronic pain during the follow-up year (either via diagnosis or opioid use). Within this category, 31% of patients (24,463) received an opioid prescription during follow-up, with 0.1% being diagnosed as opioid dependent at index ─ this proportion did not change over follow-up.

The mean age of all included patients was 64.2 years (standard deviation 13.8 years). As shown in Table 1, there were small demographic differences in the patients categorised as with and without chronic pain. Those with chronic pain appeared to be slightly older and more likely to be female.

**Table 1:** Descriptive patient characteristics at index

	Patients ‘without chronic pain’ ***n*** = 158,251	Patients ‘with chronic pain’ ***n*** = 80,055
**Sex, n (%)**		
Male	76,331 (48.2)	31,985 (40.0)
Female	81,920 (51.8)	48,070 (60.0)
**Age**		
Age, mean years, n (SD)	63.0 (13.8)	66.5 (13.5)
Min─max age, years	18─103	18─108
Age group, n (%)		
18─29	2419 (1.5)	557 (0.7)
30─39	5506 (3.5)	1594 (2.0)
40─49	17,045 (10.8)	6450 (8.1)
50─59	38,305 (24.2)	16,846 (21.0)
60─69	40,839 (25.8)	18,878 (23.6)
≥ 70	54,137 (34.2)	35,730 (44.6)

*Max* Maximum; *Min* Minimum; *SD *Standard deviation

Patients categorised as having chronic pain appeared to show a generally higher prevalence of pre-specified comorbidities (Table 2). Within each category, the proportion of patients with pre-specified comorbidities was broadly similar during the 2-year baseline and 1-year follow-up. A table of most commonly prescribed medicine classes at baseline and during the last quarter of follow-up is shown in Supplementary Table 4, Additional file 5.

**Table 2:** Prevalence of pre-specified comorbidities

Number of patients (%)	ICD-10-GM	Patients ‘without chronic pain’ ***n*** = 158,251		Patients ‘with chronic pain’ ***n*** = 80,055	
		2-yr baseline	1-yr follow-up	2-yr baseline	1-yr follow-up
**Psychiatric disorders**					
Major depressive disorder, single episode	F32*	28,501 (18.0)	25,426 (16.1)	23,155 (28.9)	21,689 (27.1)
Major depressive disorder	F32*–F33*	31,526 (20.0)	28,930 (18.3)	25,396 (31.7)	24,351 (30.4)
Major depressive disorder, recurrent	F33*	8858 (5.6)	8355 (5.3)	7974 (10.0)	7796 (9.7)
Phobic anxiety disorders	F40*	2326 (1.5)	1858 (1.2)	1692 (2.1)	1396 (1.7)
Other anxiety disorders	F41*	11,767 (7.4)	10,003 (6.3)	8986 (11.2)	7871 (9.8)
Obsessive-compulsive disorder	F42*	579 (0.4)	482 (0.3)	336 (0.4)	303 (0.4)
Reaction to severe stress, and adjustment disorders	F43*	15,912 (10.1)	11,660 (7.4)	10,857 (13.6)	8514 (10.6)
Somatoform disorders	F45*	26,226 (16.6)	20,805 (13.2)	21,377 (26.7)	20,142 (25.2)
Sleep disorders not due to a substance or known physiological condition	F51*	2635 (1.7)	2119 (1.3)	2282 (2.9)	1869 (2.3)
Abuse of non-psychoactive substances	F55*	144 (0.1)	122 (0.1)	281 (0.4)	256 (0.3)
**Metabolic disorders**					
Type 1 diabetes mellitus	E10*	3795 (2.4)	3362 (2.1)	3256 (4.1)	2819 (3.5)
Type 2 diabetes mellitus	E11*	28,745 (18.2)	28,858 (18.2)	20,393 (25.5)	20,274 (25.3)
Overweight and obesity	E66*	31,391 (19.8)	32,445 (20.5)	21,275 (26.6)	21,527 (26.9)
Disorders of lipoprotein metabolism and other lipidaemias	E78*	67,640 (42.7)	65,388 (41.3)	39,901 (49.8)	38,077 (47.6)
Elevated blood glucose level	R73*	3735 (2.4)	3105 (2.0)	2321 (2.9)	2000 (2.5)
**Cardiovascular diseases**					
Secondary hypertension	I15*	2405 (1.5)	1991 (1.3)	1788 (2.2)	1450 (1.8)
Ischemic heart diseases	I20*–I25*	29,228 (18.5)	26,245 (16.6)	21,502 (26.9)	19,446 (24.3)
Acute myocardial infarction	I21*	4801 (3.0)	4092 (2.6)	3760 (4.7)	3251 (4.1)
Atrial fibrillation and flutter	I48*	10,792 (6.8)	11,943 (7.5)	8535 (10.7)	9379 (11.7)
Non-traumatic intracranial haemorrhage/cerebral infarction	I61*–I64*	6664 (4.2)	6506 (4.1)	5207 (6.5)	5183 (6.5)
Atherosclerosis	I70*	13,671 (8.6)	14,287 (9.0)	10,520 (13.1)	10,804 (13.5)
Aortic aneurysm and dissection	I71*	3119 (2.0)	3205 (2.0)	2204 (2.8)	2248 (2.8)
Phlebitis and thrombophlebitis	I80*	7716 (4.9)	7614 (4.8)	5822 (7.3)	5623 (7.0)
**Bone and joint conditions**					
Gout	M10*	8397 (5.3)	7886 (5.0)	5377 (6.7)	5025 (6.3)
Ankylosing spondylitis	M45*	973 (0.6)	1080 (0.7)	849 (1.1)	887 (1.1)
Other inflammatory spondylopathies	M46*	1044 (0.7)	1152 (0.7)	1233 (1.5)	1242 (1.6)
Synovitis and tenosynovitis	M65*	8928 (5.6)	10,091 (6.4)	6229 (7.8)	5885 (7.4)
Osteoporosis	M80*–M81*	12,331 (7.8)	14,768 (9.3)	12,200 (15.2)	14,026 (17.5)
**Cancer**					
Malignant neoplasms of lip, oral cavity, and pharynx	C00*–C14*	547 (0.4)	448 (0.3)	443 (0.6)	388 (0.5)
Malignant neoplasms of digestive organs	C15*–C26*	6135 (3.9)	4652 (2.9)	3976 (5.0)	3356 (4.2)
Malignant neoplasms of respiratory and intrathoracic organs	C30*–C39*	1159 (0.7)	978 (0.6)	1181 (1.5)	1228 (1.5)
Malignant neoplasms of bone and articular cartilage	C40*–C41*	126 (0.1)	127 (0.1)	140 (0.2)	157 (0.2)
Melanoma and other malignant neoplasms of skin	C43*–C44*	16,953 (10.7)	12,862 (8.1)	9566 (12.0)	7156 (8.9)
Malignant neoplasms of mesothelial and soft tissue	C45*–C49*	349 (0.2)	321 (0.2)	294 (0.4)	303 (0.4)
Malignant neoplasms of breast	C50*	5512 (3.5)	4811 (3.0)	3688 (4.6)	3285 (4.1)
Malignant neoplasms of female genital organs	C51*–C58*	1665 (1.1)	1453 (0.9)	1248 (1.6)	1132 (1.4)
Malignant neoplasms of male genital organs	C60*–C63*	5351 (3.4)	5088 (3.2)	2806 (3.5)	2619 (3.3)
Malignant neoplasms of urinary tract	C64*–C68*	2631 (1.7)	2470 (1.6)	1839 (2.3)	1750 (2.2)
Malignant neoplasms of eye, brain, and other parts of central nervous system	C69*–C72*	553 (0.4)	393 (0.3)	382 (0.5)	284 (0.4)
Malignant neoplasms of thyroid and other endocrine glands	C73*–C75*	817 (0.5)	709 (0.5)	553 (0.7)	478 (0.6)
**Opioid addiction**					
Opioid dependence	F11.2	48 (< 0.1)	34 (< 0.1)	190 (0.2)	172 (0.2)
Opiate substitution	Z51.83	71 (< 0.1)	102 (0.1)	267 (0.3)	405 (0.5)

*ICD-10-GM* International Statistical Classification of Diseases and Related Health Problems, 10th edition, German modification; *yr* Year

### Healthcare resource utilisation

The observed incidence of healthcare resource utilisation during follow-up for patients with and without chronic pain is shown in Table 3. Patients categorised as having chronic pain had a substantially higher risk of needing every type of healthcare resource studied, as predicted using negative binomial regression modelling with IPTW. The RRs for hospitalisation, incapacity to work, outpatient contacts, and prescriptions for physical therapy and psychotherapy are shown in Supplementary Table 5, Additional file 6.

**Table 3:** Observed healthcare resource utilisation during the 1-year follow-up

	Patients ‘without chronic pain’ ***n*** = 158,251	Patients ‘with chronic pain’ ***n*** = 80,055
**Hospitalisations**		
≥ 1 hospitalisation, n (%)	36,991 (23.4)	30,175 (37.7)
Number of hospitalisations for all patients	54,603	55,048
Total number of inpatient days	393,247	466,086
Number of emergency hospitalisations for all patients	20,874	22,912
Length of hospitalisation, days, mean (SD)	7.2 (9.3)	8.5 (9.7)
**Outpatient physician contacts**		
≥ 1 contact, n (%)	157,888 (99.8)	79,834 (99.7)
**Incapacity to work**		
≥ 1 days incapable of working, n (%)	40,216 (25.4)	16,938 (21.2)
Days incapable of working for those with ≥1 day, mean (SD)	16.4 (39.1)	21.6 (52.8)
**Prescriptions for physical therapy**		
≥ 1 prescriptions, n (%)	63,699 (40.3)	39,615 (49.5)
Sessions for those with ≥1 prescription, mean (SD)	13.6 (11.5)	16.9 (16.4)
**Prescriptions for psychotherapy**		
≥ 1 prescriptions, n (%)	2739 (1.7)	1867 (2.3)
Sessions for those with ≥1 prescription, mean (SD)	12.0 (11.0)	11.7 (9.4)

*SD* Standard deviation

### Healthcare costs

**Fig. 2:**
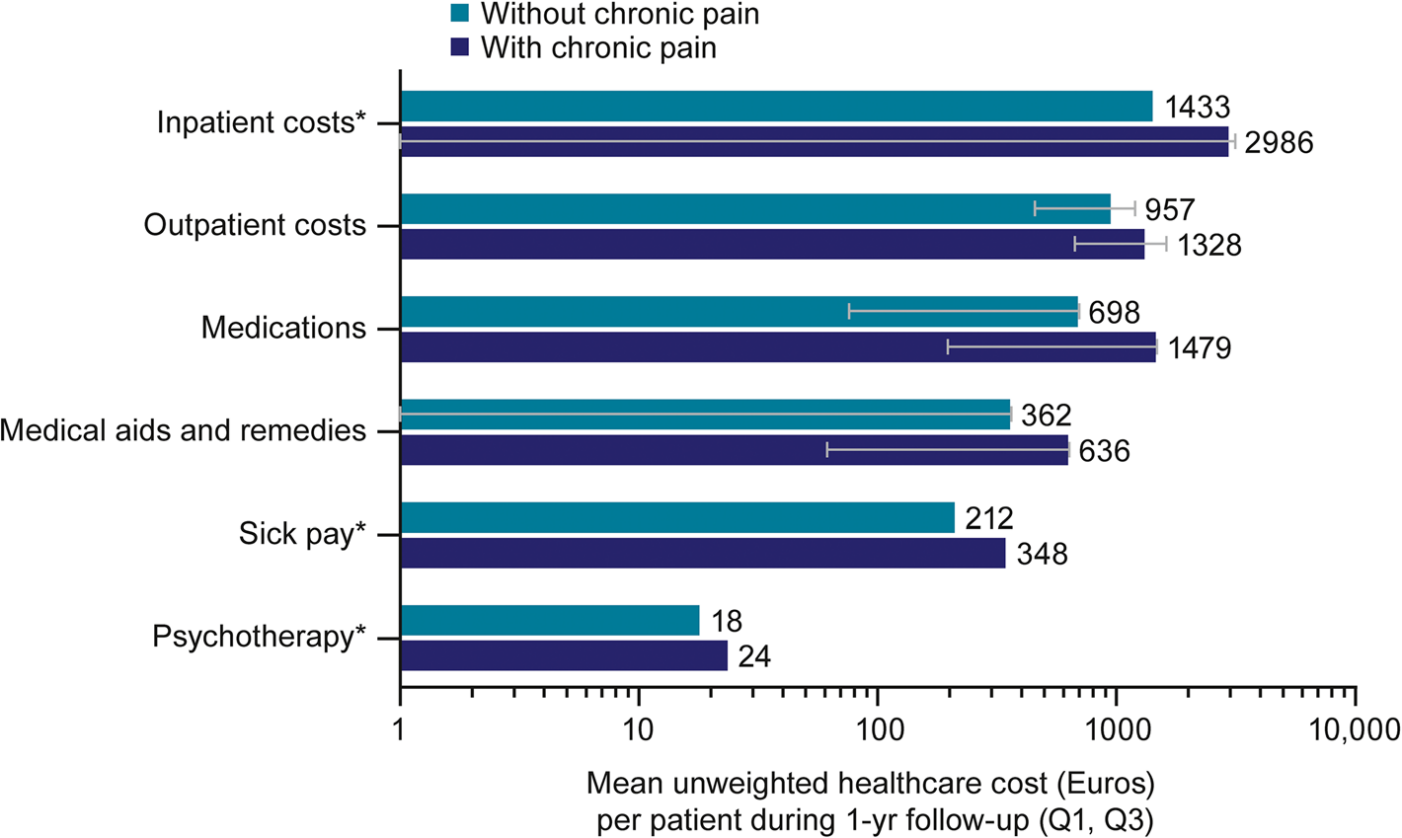
Observed healthcare costs during follow-up. *Error bars not shown where quartiles 1 and 3 are both 0. *Q* Quartile; *yr* Year

**Fig. 3:**
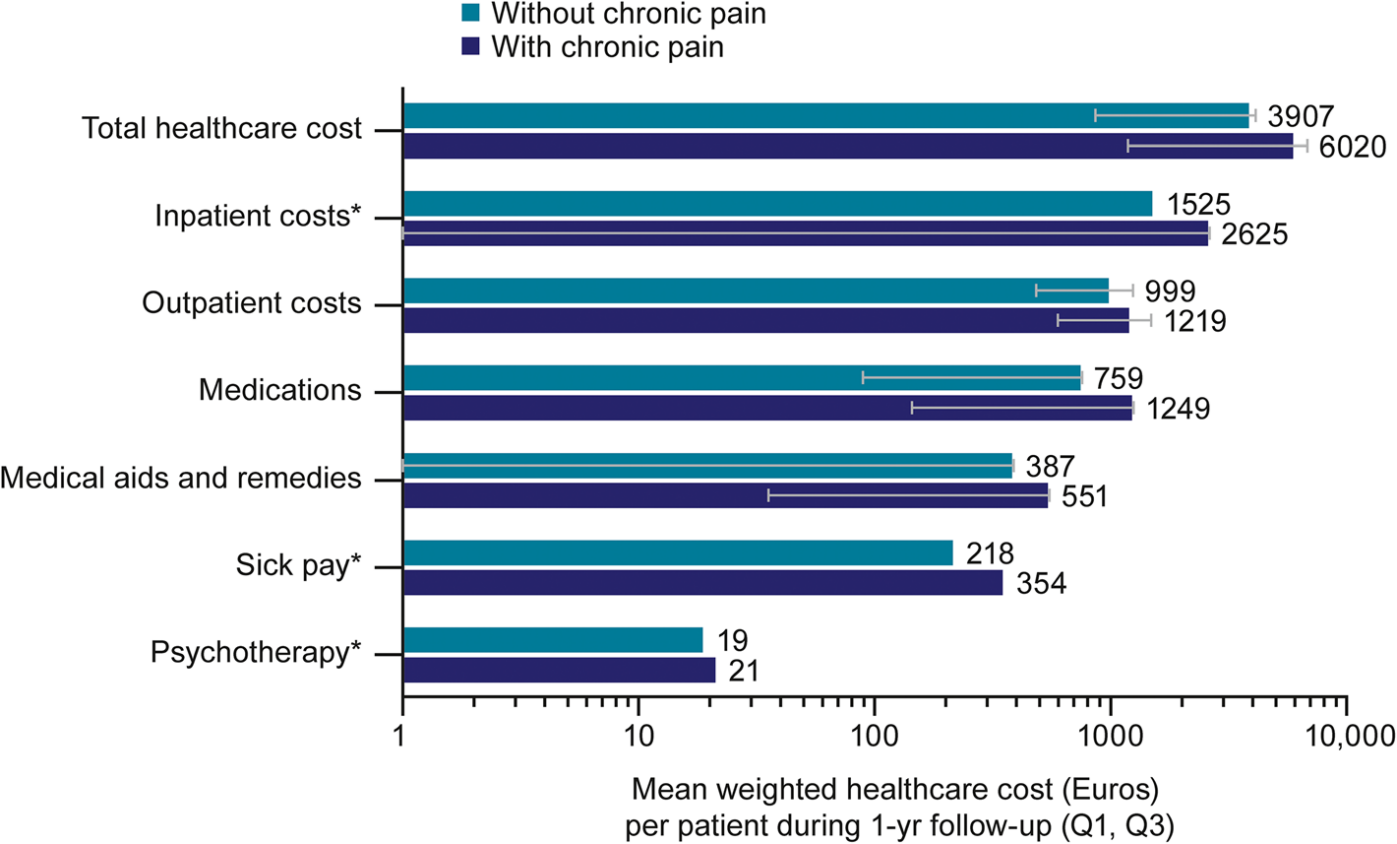
Estimated healthcare costs during follow-up based on log-linear regression with inverse probability of treatment weighting. *Error bars not shown where quartiles 1 and 3 are both 0. *Q* Quartile; *yr* Year

Total observed healthcare cost over the follow-up year in mean Euros (quartile [Q]1, Q3) were 6801 (1439, 8153) for patients categorised as with chronic pain, compared with 3682 (791, 3787) for patients without chronic pain; representing an 85% increase in total cost for patients categorised as having chronic pain. Patients with chronic pain had higher observed mean healthcare costs in all categories (Fig. 2). Hospitalisations (inpatient and outpatient) accounted for the majority of observed mean costs in both groups. Log-linear regression-based estimates for mean healthcare costs by category are shown in Fig. 3. A patient with OA and chronic pain was estimated to have 1.51 times (95% CI: 1.36, 1.66) the healthcare costs of a patient without chronic pain.

## Discussion

OA is prevalent in the German population and is more common as age increases [3]. It is a chronic condition, often associated with debilitating pain. This pain can place great strain on a patient’s life and also contributes towards the substantial overall socioeconomic impact of OA [22]. The specific cost burden of treating patients with OA and chronic pain has not yet been determined. Here we demonstrate that this treatment represents a substantial socioeconomic burden for the German healthcare system.

Using claims data from the German InGef database, we show that patients with OA and chronic pain have a higher observed utilisation of all notable healthcare resources and about 85% extra cost in the year following OA diagnosis compared with patients with OA but without chronic pain. However, our population of patients categorised as with chronic pain was slightly older and had a higher comorbidity burden than those without. To address this, we specified generalised linear models with IPTW to adjust for different demographic or comorbidity profiles. Comparable findings arose from this model, again showing a substantially higher risk of hospitalisation, outpatient physician contact, incapacity to work, and prescriptions for physical therapy and psychotherapy during the year following OA diagnosis in patients categorised as with chronic pain as compared with those without. Furthermore, an average patient with OA and chronic pain was estimated to have ~ 1.5 times higher overall annual healthcare costs in the year following OA diagnosis than a patient with OA not complicated by chronic pain. This is slightly lower than the 85% increase seen for the observed costs, probably due to the removal of bias by the model.

In this study, we defined patients as having chronic pain if they had a chronic pain diagnosis or an opioid prescription during follow-up. Data on the use of opioids in the treatment of OA pain in Germany are scarce but a 2019 meta-analysis suggested that the background prevalence of opioid prescription ranges from < 1% to ~ 6% [23]. A paper included in this meta-analysis found OA to be one of the most common diagnoses treated with opioids, and that 8.5% of weak opioid prescriptions in 2010 were for the treatment of knee OA [24]. Our findings suggest that ~ 10% of patients with painful OA received an opioid prescription in the year following diagnosis. This corresponds well with findings from a recent European survey, which found 10.6% of German patients use an opioid for the treatment of OA pain, often long term, but with poor adherence [25].

International guidelines for the non-surgical treatment of hip and knee OA have been subtly evolving over time ─ generally moving away from paracetamol and more clearly defining the situations where opioid use is appropriate [8–14]. Furthermore, guidelines increasingly recognise the need to consider comorbidities when selecting pharmacological therapy, and also to incorporate non-pharmacological therapies such as arthritis education, exercise programs, and dietary weight management [8–14]. The updated 2019 Osteoarthritis Research Society International guidelines build on previous editions by developing patient-focused treatment recommendations for individuals with knee, hip, and polyarticular osteoarthritis [8, 9]. Aiming to encourage individualised treatment decision-making, the guidelines provide treatment algorithms for patients with a range of common comorbidity combinations [8]. Within these, oral nonsteroidal anti-inflammatory drugs are not recommended for patients with cardiovascular comorbidities or frailty. Furthermore, oral or transdermal opioids are strongly recommended against for all patients with OA based on strong evidence showing a lack of efficacy and the potential risk of chemical dependency. German guidelines on the treatment of hip and knee OA recommend opioid therapy for short durations only; for example, as bridging therapy prior to joint replacement surgery [13, 14]. The German guidelines on the long-term use of opioids in non-cancer pain suggest that use in OA should be limited to situations where joint replacement surgery is not possible/not wanted and where other pharmacological/non-pharmacological therapies are unsuitable/ineffective [15]. Together, these guidelines suggest that there are limited situations where opioids are recommended for the treatment of OA; however, our findings demonstrate that a significant proportion of patients with OA are receiving opioid prescriptions in Germany.

It is clear from our findings that chronic pain is associated with substantial extra disease burden in patients with OA. While several papers have previously evaluated the cost of OA in patients taking opioids [26, 27], and we note a recent study additionally reporting on HRU and costs in patients with OA by pain severity episodes [28], we believe that our study uniquely assesses the additional burden of chronic pain as defined by opioid use or diagnosis. As the first assessment of the cost burden of chronic pain in German patients, this study provides a useful estimate of the extra cost burden of chronic pain. This study used claims data from Germany, but our findings are likely to be generalisable to other Western countries with comparable public healthcare systems.

### Limitations

The use of claims data has some inherent limitations. We acknowledge that there is potential selection bias in the InGef database, as it does not cover ~ 10% of the German population who use private medical insurance. However, the InGef database is stratified by age and gender, making it more representative of the German population and to which generalisability has been demonstrated [29]. Additionally, while our weighted model was designed to take many demographic and comorbidity factors into account, a residual amount of effect bias may remain – for example, if chronic pain is linked to more severe OA. Subtle changes in the construction of case weights (e.g. by considering those substances with the highest mean cost per patient rather than the most frequently prescribed substances) may also alter the reported HRU and cost estimates, but alternative model specifications were not explored. Furthermore, any statistical adjustment made to our findings may incur collider bias, where an outcome is influenced by more than one variable i.e. HRU and costs are necessarily defined by direct and indirect components. As in all analyses of claims data, it is a limitation that we were unable to causally link diagnoses and prescriptions, therefore, we are unable to be sure if all patients with chronic pain were captured, or if perhaps some patients were taking opioids to address pain related to a comorbidity. Accordingly, our method to identify patients with chronic pain was likely robust but not guaranteed to be free of measurement bias. Furthermore, measurement bias may arise from inaccurately coding the database.

Our use of claims data means that we cannot determine the indication for which each drug was selected. It also means that non-prescription drug use was not captured, including over-the-counter medication, traditional medicines, and lifestyle adaptations. A representative survey of German patients with OA in 2014 noted significant use (~ 80%) of self-paid services, suggesting that a significant proportion of OA treatment might not be captured in claims data [30]. How much of this is related to pain is unknown; while opioid analgesic treatments are very unlikely to be used without clinical supervision, patients might still be using available over-the-counter medication to treat their pain. Because of the structure of the data, this study could not evaluate personal ‘costs’ of advanced OA, such as a reduction in quality of life and effects on mental health.

## Conclusions

The burden of disease in patients with OA is substantially higher in those who also experience chronic pain. This burden was visible as higher HRU across hospitalisations, outpatient physician contacts, incapacity to work, and prescriptions for physical therapy or psychotherapy, and also as higher associated healthcare costs. To reduce the burden of disease for the individual and for society, new ways to optimise the prevention and treatment of chronic pain in OA are required.

## Supplementary Information

**Additional file 1: Supplementary Table 1.** Definitions for health resource utilisation outcomes and healthcare costs. Definitions used for the assessment of health resource utilisation outcomes and healthcare costs.

**Additional file 2: Supplementary Table 2.** Logistic regression coefficients for variables contributing to the propensity score. Factors incorporated into the logistic regression model to estimate the individual probability of a patient being classified as ‘with chronic pain’. Due to the operational definition of pain in this study, pain medications were excluded from the set of assessed factors. *CI* 95% confidence interval

**Additional file 3: Supplementary Table 3.** Propensity score distribution by percentile. Patients in each percentile of the propensity score.

**Additional file 4: Supplementary Figure 1.** Propensity score distribution. Percentage of patients with each propensity score.

**Additional file 5: Supplementary Table 4.** Most frequently prescribed drugs . Top 10 most frequently prescribed drug classes (defined by the Anatomical Therapeutic Chemical Classification System) for the two years prior to index (baseline) and the last quarter of the follow-up year for patients with osteoarthritis, classified as with and without chronic pain. Defined by Anatomical Therapeutic Chemical Classification System. *yr* Year

**Additional file 6: Supplementary Table 5.** Estimated healthcare resource utilisation in 2016 based on negative binomial regression with inverse probability of treatment weighting. Conditional means and rate ratios (both with 95% confidence intervals) for healthcare resource utilisation for patients with osteoarthritis, classified as with and without chronic pain in the year following index, estimated using negative binomial regression with inverse probability of treatment weighting. All are per patient-year. *p < 0.001; †*p* < 0.05 by chi squared with Wald test. *95%* *CI* 95% confidence interval

## Data Availability

The data that support the findings of this study are available from InGef but restrictions apply to the availability of these data, which were used under license for the current study and are not publicly available. Data are available from the authors upon reasonable request and with permission of InGef.

## Abbreviations

ATC: Anatomical Therapeutic Chemical Classification System
CI: Confidence interval
HRU: Healthcare resource utilisation
ICD-10-GM: International Statistical Classification of Diseases and Related Health Problems, 10th edition, German modification
InGef: Institut für Angewandte Gesundheitsforschung
IPTW: Inverse probability of treatment weighting
OA: Osteoarthritis
Q: Quartile
RR: Rate ratio
SD: Standard deviation
yr: Year
