# Supplementary material for: The burden of chronic pain for patients with osteoarthritis in Germany: a retrospective cohort study of claims data
Source: BMC Musculoskelet Disord. 2021 Mar 31;22:317. doi: 10.1186/s12891-021-04180-1 (PMC8011414; doi:10.1186/s12891-021-04180-1)
Supplement: Supplementary file 1 — Additional file 1: Supplementary Table 1. Definitions for health resource utilisation outcomes and healthcare costs. Definitions used for the assessment of health resource utilisation outcomes and healthcare costs. [file 12891_2021_4180_MOESM1_ESM.docx]

**Supplementary Table 1:** Definitions for health resource utilisation outcomes and healthcare costs

| **Outcome** | **Definition** |
| --- | --- |
| **Health resource utilisation** | |
| Hospitalisations | Admission reasons ‘Krankenhausbehandlung, vollstationär’ or ‘Krankenhausbehandlung vollstationär mit vorausgegangener vorstationärer Behandlung’.  Presented as number of patients (%) with at least 1 hospitalisation, total number of hospitalisations for all patients in the group, total number of hospitalization days for the group, the number of patients with emergency hospitalisations all patients in the group (admission code: “Notfall”), and mean duration of hospitalisations (days with standard deviation). |
| Outpatient physician contacts | Each physician contact was defined by a distinct date and a recorded physician service.  Presented as the number of patients with at least 1 contact (%). |
| Incapacity to work | Based on the first and last day of incapacity recorded in the database. Consecutive episodes are summed.  Presented as the number of patients (%) with any days, and, for these patients, mean days with standard deviation. |
| Prescriptions for physical therapy | Identified as remedy codes WS1 or 2 (spinal cord disorders; short and long term), EX1, 2, or 3 (injuries and disorders of the extremities and pelvis; short, medium and long term), or CS (chronic pain syndrome).  Presented as number of patients (%) with any prescriptions, and, for these patients, the mean number of prescribed sessions with standard deviation. |
| Prescriptions for psychotherapy | Identified using ‘Gebührenordnungsposition’ codes, corresponding to short and long term, individual and long-group incidences of cognitive behavioural therapy, psychoanalysis, or behavioural therapy.  Presented as number of patients (%) with any prescriptions, and, for these patients, the mean number of prescribed sessions with standard deviation. |
| **Healthcare costs**  *All presented as mean Euros with quartile 1 and quartile 3* | |
| Inpatient and outpatient costs | The total costs of hospitalisations, including any additional payments made by the patient.  Outpatient costs (excluding psychotherapy) are fixed reimbursements plus the sum of points multiplied by the current valid point value plus dialysis costs. |
| Medications | Gross amount reimbursed. Does not include pharmacy or manufacturing costs, or patient co-payments. |
| Medical aids and remedies | Reimbursement for medical aids (‘Hilfsmittel’, e.g. crutches, wheelchairs, catheters) and remedies (‘Heilmittel’, e.g. physical therapy, speech therapy). |
| Sick pay | Total amount paid. |
| Psychotherapy | Summed fixed reimbursements. |
