# Supplementary material for: The burden of chronic pain for patients with osteoarthritis in Germany: a retrospective cohort study of claims data
Source: BMC Musculoskelet Disord. 2021 Mar 31;22:317. doi: 10.1186/s12891-021-04180-1 (PMC8011414; doi:10.1186/s12891-021-04180-1)
Supplement: Supplementary file 2 — Additional file 2: Supplementary Table 2. Logistic regression coefficients for variables contributing to the propensity score. Factors incorporated into the logistic regression model to estimate the individual probability of a patient being classified as ‘with chronic pain’. Due to the operational definition of pain in this study, pain medications were excluded from the set of assessed factors. CI 95% confidence interval [file 12891_2021_4180_MOESM2_ESM.docx]

**Supplementary Table 2:** Logistic regression coefficients for variables contributing to the propensity score

| **Variable** | **ß(SE)** | **Odds Ratio** | **Lower CI** | **Upper CI** | **p** |
| --- | --- | --- | --- | --- | --- |
| Intercept | -2.2865 (0.0330) | . | . | . | <.0001 |
| Age | 0.0120 (0.0005) | 1.01 | 1.01 | 1.01 | <.0001 |
| Sex | -0.2355 (0.0115) | 0.79 | 0.77 | 0.81 | <.0001 |
| Type of osteoarthritis (knee vs. hip) | -0.1575 (0.0097) | 0.85 | 0.84 | 0.87 | <.0001 |
| Essential (primary) hypertension | 0.0583 (0.0123) | 1.06 | 1.03 | 1.09 | <.0001 |
| Dorsalgia | 0.2149 (0.0105) | 1.24 | 1.21 | 1.27 | <.0001 |
| Disorders of refraction and accommodation | -0.0547 (0.0105) | 0.95 | 0.93 | 0.97 | <.0001 |
| Disorders of lipoprotein metabolism and other lipidemias | -0.0316 (0.0106) | 0.97 | 0.95 | 0.99 | 0.0028 |
| Encounter for screening for malignant neoplasms | -0.1052 (0.0104) | 0.90 | 0.88 | 0.92 | <.0001 |
| Encounter for general exam without complaint, suspected or reported diagnoses | -0.0532 (0.0098) | 0.95 | 0.93 | 0.97 | <.0001 |
| Spondylosis | 0.1855 (0.0115) | 1.20 | 1.18 | 1.23 | <.0001 |
| Need for immunization against other single viral diseases | 0.0533 (0.0111) | 1.05 | 1.03 | 1.08 | <.0001 |
| Major depressive disorder (single episode) | 0.2861 (0.0113) | 1.33 | 1.30 | 1.36 | <.0001 |
| Overweight and obesity | 0.1092 (0.0117) | 1.12 | 1.09 | 1.14 | <.0001 |
| Thoracic, thoracolumbar, and lumbosacral intervertebral disc disorders | 0.3951 (0.0125) | 1.48 | 1.45 | 1.52 | <.0001 |
| Other and unspecified dorsopathies (not elsewhere classified) | 0.1466 (0.0119) | 1.16 | 1.13 | 1.19 | <.0001 |
| Acute upper respiratory infections of multiple and unspecified sites | 0.1333 (0.0116) | 1.14 | 1.12 | 1.17 | <.0001 |
| Type 2 diabetes mellitus | 0.0772 (0.0159) | 1.08 | 1.05 | 1.11 | <.0001 |
| Menopausal and other perimenopausal disorders | -0.0669 (0.0136) | 0.94 | 0.91 | 0.96 | <.0001 |
| Abdominal and pelvic pain | 0.0054 (0.0121) | 1.01 | 0.98 | 1.03 | 0.6558 |
| Biomechanical lesions (not elsewhere classified) | 0.0398 (0.0124) | 1.04 | 1.02 | 1.07 | 0.0014 |
| Other joint disorder (not elsewhere classified) | 0.0616 (0.0119) | 1.06 | 1.04 | 1.09 | <.0001 |
| Gastritis and duodenitis | 0.0207 (0.0131) | 1.02 | 1.00 | 1.05 | 0.1143 |
| Other and unspecified soft tissue disorders (not elsewhere classified) | 0.2291 (0.0127) | 1.26 | 1.23 | 1.29 | <.0001 |
| Other disorders of urinary system | 0.0687 (0.0131) | 1.07 | 1.04 | 1.10 | <.0001 |
| Gastro-esophageal reflux disease | 0.0084 (0.0132) | 1.01 | 0.98 | 1.03 | 0.5233 |
| Encounter for other specific examination without complaint, suspected or reported diagnoses | -0.0426 (0.0126) | 0.96 | 0.94 | 0.98 | 0.0007 |
| Presence of other functional implants | 0.1553 (0.0143) | 1.17 | 1.14 | 1.20 | <.0001 |
| Chronic ischemic heart disease | 0.0928 (0.0148) | 1.10 | 1.07 | 1.13 | <.0001 |
| Other and unspecified osteoarthritis | 0.1081 (0.0129) | 1.11 | 1.09 | 1.14 | <.0001 |
| Varicose veins of lower extremities | -0.0440 (0.0124) | 0.96 | 0.93 | 0.98 | 0.0004 |
| Other cataract | 0.0089 (0.0142) | 1.01 | 0.98 | 1.04 | 0.53 |
| Other nontoxic goiter | -0.0620 (0.0129) | 0.94 | 0.92 | 0.96 | <.0001 |
| Other disorders of external ear | 0.0029 (0.0125) | 1.00 | 0.98 | 1.03 | 0.817 |
| Ibuprofen | 0.1782 (0.0099) | 1.20 | 1.17 | 1.22 | <.0001 |
| Metamizole sodium | 0.4920 (0.0106) | 1.64 | 1.60 | 1.67 | <.0001 |
| Pantoprazole | 0.2269 (0.0113) | 1.25 | 1.23 | 1.28 | <.0001 |
| Diclofenac | 0.1597 (0.0112) | 1.17 | 1.15 | 1.20 | <.0001 |
| Simvastatin | -0.0058 (0.0140) | 0.99 | 0.97 | 1.02 | 0.6758 |
| Levothyroxine sodium | 0.0482 (0.0129) | 1.05 | 1.02 | 1.08 | 0.0002 |
| Ramipril | 0.0003 (0.0129) | 1.00 | 0.98 | 1.03 | 0.9822 |
| Omeprazole | 0.2084 (0.0142) | 1.23 | 1.20 | 1.27 | <.0001 |
| Metoprolol | 0.0003 (0.0146) | 1.00 | 0.97 | 1.03 | 0.9809 |
| Bisoprolol | -0.0207 (0.0144) | 0.98 | 0.95 | 1.01 | 0.1504 |
| Amlodipine | -0.0024 (0.0144) | 1.00 | 0.97 | 1.03 | 0.8652 |
| Cefuroxime | 0.1686 (0.0142) | 1.18 | 1.15 | 1.22 | <.0001 |
| Amoxicillin | 0.1721 (0.0139) | 1.19 | 1.16 | 1.22 | <.0001 |
| Prednisolone | 0.2722 (0.0151) | 1.31 | 1.27 | 1.35 | <.0001 |
| Ciprofloxacin | 0.1110 (0.0149) | 1.12 | 1.09 | 1.15 | <.0001 |
| Torasemide | 0.2594 (0.0170) | 1.30 | 1.25 | 1.34 | <.0001 |
| Imidazoles/triazoles in combination with corticosteroids | 0.1098 (0.0194) | 1.12 | 1.07 | 1.16 | <.0001 |
| Enoxaparin | 0.1317 (0.0201) | 1.14 | 1.10 | 1.19 | <.0001 |
| Salbutamol | 0.2297 (0.0167) | 1.26 | 1.22 | 1.30 | <.0001 |
| Acetylsalicylic acid | 0.1192 (0.0182) | 1.13 | 1.09 | 1.17 | <.0001 |
| Candesartan | -0.0041 (0.0197) | 1.00 | 0.96 | 1.04 | 0.8368 |
| Dexamethasone and anti-infectives | 0.0044 (0.0167) | 1.00 | 0.97 | 1.04 | 0.7913 |
| Allopurinol | 0.0782 (0.0172) | 1.08 | 1.05 | 1.12 | <.0001 |
| Metformin | 0.0009 (0.0202) | 1.00 | 0.96 | 1.04 | 0.9655 |
| Metoclopramide | 0.3372 (0.0184) | 1.40 | 1.35 | 1.45 | <.0001 |
| [Doxycycline](https://www.whocc.no/atc_ddd_index/?code=J01AA02&showdescription=yes) | 0.1209 (0.0176) | 1.13 | 1.09 | 1.17 | <.0001 |
| Clindamycin | 0.1168 (0.0182) | 1.12 | 1.08 | 1.16 | <.0001 |
| Hydrochlorothiazide | 0.0443 (0.0194) | 1.05 | 1.01 | 1.09 | 0.0225 |
| Azithromycin | 0.2216 (0.0190) | 1.25 | 1.20 | 1.30 | <.0001 |
| Glucose | 0.1300 (0.0228) | 1.14 | 1.09 | 1.19 | <.0001 |

*CI* 95% confidence interval
