# Supplementary material for: The burden of chronic pain for patients with osteoarthritis in Germany: a retrospective cohort study of claims data
Source: BMC Musculoskelet Disord. 2021 Mar 31;22:317. doi: 10.1186/s12891-021-04180-1 (PMC8011414; doi:10.1186/s12891-021-04180-1)
Supplement: Supplementary file 3 — Additional file 3: Supplementary Table 3. Propensity score distribution by percentile. Patients in each percentile of the propensity score. [file 12891_2021_4180_MOESM3_ESM.docx]

**Supplementary Table 3:** Propensity score distribution by percentile

|  | **Categorised as ‘with chronic pain’** | | | | **Total** | |
| --- | --- | --- | --- | --- | --- | --- |
|  | **No** | | **Yes** | |  |  |
| **Percentile** | **n** | **%** | **n** | **%** | **n** | **%** |
| 0.05-0.10 | 1512 | 0.96 | 154 | 0.19 | 1666 | 0.70 |
| 0.10-0.15 | 20,128 | 12.72 | 2838 | 3.55 | 22,966 | 9.64 |
| 0.15-0.20 | 29,618 | 18.72 | 6516 | 8.14 | 36,134 | 15.16 |
| 0.20-0.25 | 26,193 | 16.55 | 8173 | 10.21 | 34,366 | 14.42 |
| 0.25-0.30 | 20,950 | 13.24 | 8204 | 10.25 | 29,154 | 12.23 |
| 0.30-0.35 | 15,899 | 10.05 | 7994 | 9.99 | 23,893 | 10.03 |
| 0.35-0.40 | 12,299 | 7.77 | 7559 | 9.44 | 19,858 | 8.33 |
| 0.40-0.45 | 9370 | 5.92 | 6752 | 8.43 | 16,122 | 6.77 |
| 0.45-0.50 | 6961 | 4.40 | 6403 | 8.00 | 13,364 | 5.61 |
| 0.50-0.55 | 5249 | 3.32 | 5879 | 7.34 | 11,128 | 4.67 |
| 0.55-0.60 | 3760 | 2.38 | 5157 | 6.44 | 8917 | 3.74 |
| 0.60-0.65 | 2701 | 1.71 | 4343 | 5.43 | 7044 | 2.96 |
| 0.65-0.70 | 1724 | 1.09 | 3679 | 4.60 | 5403 | 2.27 |
| 0.70-0.75 | 1056 | 0.67 | 2784 | 3.48 | 3840 | 1.61 |
| 0.75-0.80 | 537 | 0.34 | 1900 | 2.37 | 2437 | 1.02 |
| 0.80-0.85 | 232 | 0.15 | 1124 | 1.40 | 1356 | 0.57 |
| 0.85-0.90 | 56 | 0.04 | 484 | 0.60 | 540 | 0.23 |
| 0.90-0.95 | 6 | 0.00 | 112 | 0.14 | 118 | 0.05 |
| **Total** | 158,251 | 100.00 | 80,055 | 100.00 | 238,306 | 100.00 |
