# Supplementary figures and images for: The burden of chronic pain for patients with osteoarthritis in Germany: a retrospective cohort study of claims data
Source: BMC Musculoskelet Disord. 2021 Mar 31;22:317. doi: 10.1186/s12891-021-04180-1 (PMC8011414; doi:10.1186/s12891-021-04180-1)

## Supplementary Figure 1. Propensity score distribution

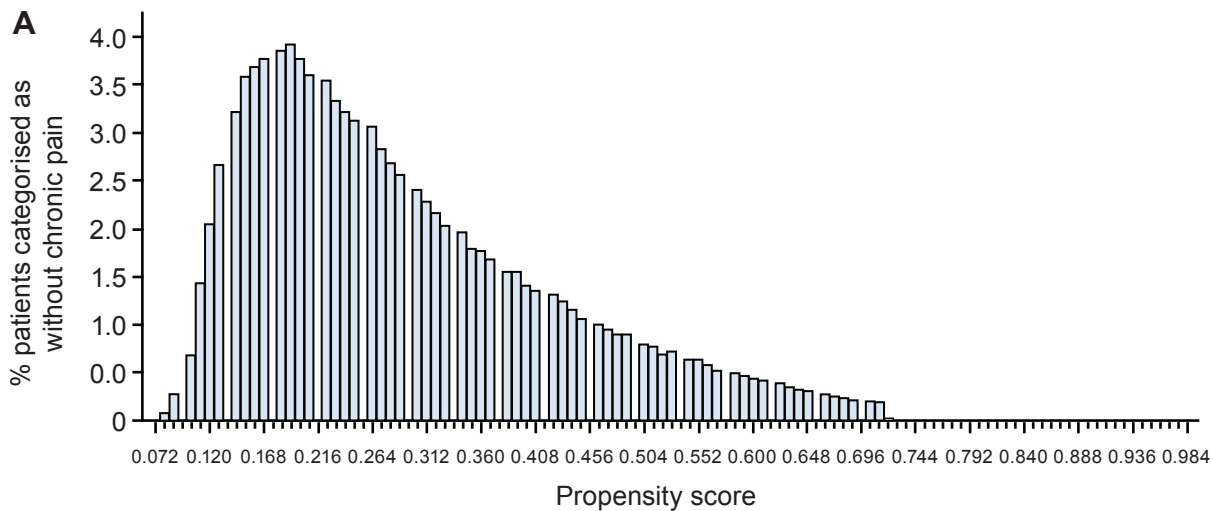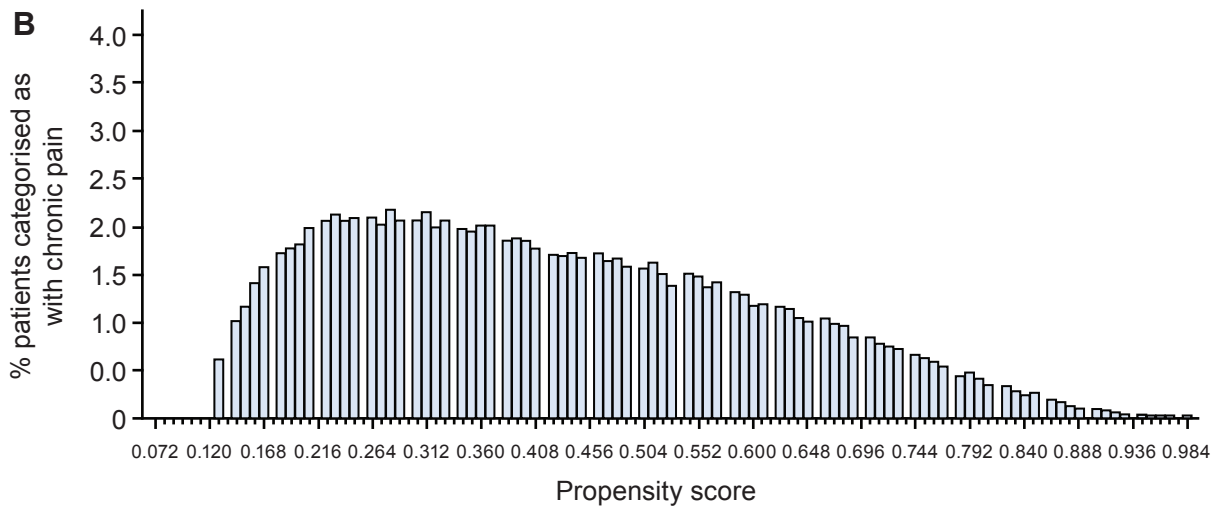

Supplement: Supplementary file 4 — Additional file 4: Supplementary Figure 1. Propensity score distribution. Percentage of patients with each propensity score. [file 12891_2021_4180_MOESM4_ESM.pdf]
