# Supplementary material for: The burden of chronic pain for patients with osteoarthritis in Germany: a retrospective cohort study of claims data
Source: BMC Musculoskelet Disord. 2021 Mar 31;22:317. doi: 10.1186/s12891-021-04180-1 (PMC8011414; doi:10.1186/s12891-021-04180-1)
Supplement: Supplementary file 5 — Additional file 5: Supplementary Table 4. Most frequently prescribed drugs . Top 10 most frequently prescribed drug classes (defined by the Anatomical Therapeutic Chemical Classification System) for the two years prior to index (baseline) and the last quarter of the follow-up year for patients with osteoarthritis, classified as with and without chronic pain. Defined by Anatomical Therapeutic Chemical Classification System. yr Year [file 12891_2021_4180_MOESM5_ESM.docx]

**Supplementary Table 4:** Most frequently prescribed drugs

| **Number of patients (%)** | **Patients  ‘without chronic pain’**  ***n* = 158,251** | | **Patients  ‘with chronic pain’**  ***n* = 80,055** | |
| --- | --- | --- | --- | --- |
| **Rank** | **2-yr baseline** | **Last quarter of follow-up** | **2-yr baseline** | **Last quarter of follow-up** |
| 1 | Anti-inflammatory and anti-rheumatic products  78,620 (50) | Agents acting on the renin-angiotensin system  50,328 (32) | Anti-inflammatory and anti-rheumatic products  51,134 (64) | Agents acting on the renin-angiotensin system  30,774 (38) |
| 2 | Anti-bacterials for systemic use  76,923 (49) | Beta blocking agents  33,072 (21) | Anti-bacterials for systemic use  49,800 (62) | Analgesics  22,184 (28) |
| 3 | Agents acting on the renin-angiotensin system  66,340 (42) | Thyroid therapeutics  23,691 (15) | Analgesics  49,284 (62) | Drugs for acid related disorders  21,760 (27) |
| 4 | Drugs for acid-related disorders  51,869 (33) | Drugs for acid-related disorders  22,750 (14) | Drugs for acid-related disorders  41,951 (52) | Beta blocking agents  21,569 (27) |
| 5 | Beta blocking agents  46,075 (29) | Anti-inflammatory and anti-rheumatic products  22,620 (14) | Agents acting on the renin-angiotensin system  41,931 (52) | Anti-inflammatory and anti-rheumatic products  17,766 (22) |
| 6 | Analgesics  36,329 (23) | Lipid-modifying agents  21,832 (14) | Beta blocking agents  30,342 (38) | Thyroid therapeutics  14,889 (19) |
| 7 | Lipid-modifying agents  33,973 (21) | Calcium channel blockers  16,907 (11) | Lipid-modifying agents  22,923 (29) | Lipid-modifying agents  14,320 (18) |
| 8 | Thyroid therapeutics  31092 (20) | Anti-thrombotic agents  16,872 (11) | Anti-thrombotic agents  22,510 (28) | Anti-thrombotic agents  13,362 (17) |
| 9 | Ophthalmologicals  28,983 (18) | Anti-bacterials for systemic use  16,053 (10) | Diuretics  20,420 (26) | Diuretics  13,255 (17) |
| 10 | Anti-thrombotic agents  28,797 (18) | Diuretics  14,724 (9) | Thyroid therapeutics  19,842 (25) | Anti-bacterials for systemic use  12,126 (15) |

Defined by Anatomical Therapeutic Chemical Classification System. *yr* Year
