# Supplementary material for: The burden of chronic pain for patients with osteoarthritis in Germany: a retrospective cohort study of claims data
Source: BMC Musculoskelet Disord. 2021 Mar 31;22:317. doi: 10.1186/s12891-021-04180-1 (PMC8011414; doi:10.1186/s12891-021-04180-1)
Supplement: Supplementary file 6 — Additional file 6: Supplementary Table 5. Estimated healthcare resource utilisation in 2016 based on negative binomial regression with inverse probability of treatment weighting. Conditional means and rate ratios (both with 95% confidence intervals) for healthcare resource utilisation for patients with osteoarthritis, classified as with and without chronic pain in the year following index, estimated using negative binomial regression with inverse probability of treatment weighting. All are per patient-year. *p < 0.001; †p < 0.05 by chi squared with Wald test. 95% CI 95% confidence interval [file 12891_2021_4180_MOESM6_ESM.docx]

**Supplementary Table 5:** Estimates of healthcare resource utilisation in 2016 based on negative binomial regression with inverse probability of treatment weighting

|  | **Conditional mean (95% CI)** | | **Rate ratio (95% CI)**  **for patients with chronic pain vs those without** |
| --- | --- | --- | --- |
|  | **Patients ‘without chronic pain’** | **Patients ‘with chronic pain’** |  |
| **Hospitalisations** | | | |
| Number of hospitalisations | 0.35 (0.34, 0.35) | 0.65 (0.63, 0.66) | 1.84 (1.81, 1.87)* |
| Length of hospitalisations, days | 6.87 (6.77, 6.97) | 7.92 (7.56, 8.08) | 1.15 (1.14, 1.17)* |
| Number of emergency hospitalisations | 0.58 (0.56, 0.59) | 0.762 (0.74, 0.79) | 1.34 (1.31, 1.37)* |
| **Outpatient physician contacts** | | | |
| Number of contacts | 21.91 (21.80, 22.02) | 26.75 (26.56, 26.94) | 1.22 (1.21, 1.22)* |
| **Incapacity to work** | | | |
| Incapacity to work, days | 10.43 (10.04, 10.82) | 15.27 (14.49, 16.10) | 1.37 (1.32, 1.42)* |
| **Prescriptions for physical therapy** | | | |
| Number of prescriptions | 1.03 (1.01, 1.04) | 1.50 (1.42, 1.48) | 1.38 (1.36, 1.40)* |
| **Prescriptions for psychotherapy** | | | |
| Number of prescriptions | 0.22 (0.20, 0.25) | 0.25 (0.21, 0.29) | 1.13 (1.00, 1.27)† |

*All are per patient-year*. **p*<0.001; †*p*<0.05 by chi squared with Wald test. *95% CI* 95% confidence interval
